# Induction of Cell Activation Processes by Low Frequency Electromagnetic Fields

**DOI:** 10.1100/tsw.2004.174

**Published:** 2004-10-20

**Authors:** Myrtill Simkó

**Affiliations:** University of Rostock, Institute of Cell Biology and Biosystems Technology, Division of Environmental Physiology, Albert-Einstein-Str. 3, D-18059 Rostock, Germany

**Keywords:** ELF-EMF, cytotoxicity, cell cycle control, signal transduction, cell activation, carcinogenesis, mechanisms

## Abstract

Electromagnetic fields (EMF) such as those from electric power transmission and distribution lines (50/60 Hz) have been associated with increased risk of childhood leukemia, cancer of the nervous system, and lymphomas. Several *in vitro* studies on EMF effects were performed to clarify the existing controversies, define the risks, and determine the possible mechanisms of adverse effects. In some of these reports, the effects were related to other mechanisms of carcinogenesis. Modification in cell proliferation was observed after EMF exposure and a few reports on cytotoxic effects have also been published. This limited review gives an overview of the current results of scientific research regarding *in vitro* studies on the effects of power line frequency EMF, but also cell biological mechanisms and their potential involvement in genotoxicity and cytotoxicity are discussed. Cell cycle control and signal transduction processes are included to elucidate the biochemical background of possible interactions. Exposure to EMF has been also linked to the incidence of leukemia and other tumors in some epidemiological studies and is considered as “possibly carcinogenic to humans”, but there is no well-established biological mechanism that explains such a relation. Furthermore, EMF is also shown as a stimulus for immune relevant cells (e.g., macrophages) to release free radicals. It is known that chronic activation of macrophages is associated with the onset of phagocytosis and leads to increased formation of reactive oxygen species, which themselves may cause DNA damage and are suggested to lead to carcinogenesis. To demonstrate a possible interaction between EMF and cellular systems, we present a mechanistic model describing cell activation as a major importance for cellular response.

**ABBREVIATIONS:** micronuclei (MN), cyclin dependent kinase (Cdk), cyclin dependent kinase inhibitor (CKI), inhibitor of Cdk4 (INK4), receptor tyrosine kinase (RTK), retinoblastoma protein (Rb), transcription factor (E2F), protein kinase C (PKC), reactive oxygen species (ROS), reactive nitrogen species (RNS), mitogen-activated protein kinase (MAPK), benzo(a)pyrene (BP)

*Tel.: ++49/381/498 1935/Fax: ++49/381/498 1918
©2004 with author.

### INTRODUCTION

#### General Overview

There is a controversial discussion if electromagnetic fields (EMFs) induce biological effects that might be harmful to human health. Although an increasing number of reports have been published about biological effects caused by power line frequency EMFs (50/60 Hz), there is still need for more basic research in this area because the knowledge of the underlying molecular mechanisms will allow the estimation of potential health risks. It is generally assumed that multiplicities of different primary reactions are involved in the observed cellular responses[1,2]. The question — whether EMFs cause neoplastic development leading to carcinogenesis — is of special interest.

Environmental EMFs such as those from electric power transmission and distribution lines have been associated with an increased risk of childhood leukemia, cancer of the nervous system, and lymphomas as shown in several epidemiological studies[1,2,3,4,5,6,7]. There is also a great interest in a possible association with breast cancer, in part, because breast cancer risk is substantially higher in industrialized countries than in other areas, and electric power generation and consumption is one of the hallmarks of industrialized societies. In 1987, Stevens[8] proposed a biological mechanism whereby two products of electric power generation, EMF and light at night, might contribute to mammary carcinogenesis through inhibition of melatonin. The biological plausibility of an association between EMF and breast cancer, coupled with data from occupational studies and unexplained high-incidence rates of breast cancer, suggests that further investigation of this possible association is warranted[9].

Several *in vitro* studies on EMF effects were performed to explain and determine the possible mechanism of cancer development. In some of these reports, the effects were related to other known mechanisms of carcinogenesis so that EMFs seem not to be considered as initiators of cancer. It is assumed in another line of evidence that EMFs act as cocarcinogens, whereas the causal relationship is still unclear. The findings published in these reports initiated controversial discussions about the biological effectiveness of EMF. Fundamental processes, such as alterations in DNA and RNA synthesis rates caused by EMF, were described[10,11,12], while possible direct genomic interactions with electric or magnetic fields are generally held to be unlikely. However, it is known that indirectly acting carcinogens can cause genotoxic effects as well.

Interactions occurring at or near the cell surface have been discussed[13] and the modulation of critical events in receptor-mediated signal transduction and the activation of tyrosine kinases and protein kinase C (PKC) has also been considered and described[14,15,16,17,18].

Time-varying magnetic fields can induce electric current in the body, according to Faraday's law, but the field strength within the cell and cellular components are not clear. Therefore, discrepancies of the observed data are considered to be due to differences in the cell types with different cell shapes used (such as transformed- or nontransformed cells, monolayer or suspension cell culture, human- or nonhuman cell lines, etc.), which can result in cell shape–dependent intracellular-induced current, but also due to exposure duration (from hours to days) and exposure conditions (e.g., orientation of electric- and/or magnetic fields, frequency, intensity, etc.), as well. The magnitude of the induced electric fields and the induced current densities can vary depending on the size of the cell culture dish and/or height of the filled medium. Therefore, it is important to characterize physical parameters regarding electric and magnetic fields.

Quantification of induced electric current ($J_{max}$) and electric fields ($E_{max}$) is important in evaluation and interpretation of results of *in vitro* laboratory studies; however these can be calculated as followed:

$$E_{max} = \pi \cdot f \cdot r \cdot B_0$$

where $E_{max}$ = maximum electric field (V/m), f = frequency (Hz), r = radius of dish (m), and $B_0$ = magnetic flux density (T).

The induced current density ($J_{max}$) can be calculated as

$$J_{max} = \sigma \cdot E_{max}$$

where $J_{max}$ = maximal induced current intensity ($A/m^2$) and $\sigma$ = conductivity (S/m).

The large numbers of publications from studies of whole organisms down to the subcellular level report different and very often contradictory results. Many negative findings have been reported as well. However, relevant existing data have been compiled and evaluated by the NIEHS[19] and later by IARC[20], leading to the conclusion that power line frequency EMF is classified as a possible carcinogen class 2B, which is primarily based on the increased risk for leukemia and brain tumors and living close to power lines, and on occupational exposure.

In order to clarify the question of a possible correlation between cancer and EMF, in the present review, cytotoxic and genotoxic effects were summarized. To elucidate the potential underlying mechanisms of EMF effects, experiments modulating cell cycle progression and signal transduction pathways were described at the cellular and molecular level. We also show a direct activation of cells of the immune system by EMF, which is a substantial contribution to elucidate the mechanism by which EMF interacts with cellular systems. Moreover, we studied the carcinogenic potential of EMF as an initiator or a promoter and discuss different biophysical mechanisms. Furthermore, we propose a unified hypothesis for the interaction between EMF and biological systems, which may have relevance for therapeutic applications of EMF for cell activation, and provide a way of linking EMF with carcinogenesis.

### *In Vitro* Studies on the Effects of Electromagnetic Fields

There are several indications of cellular responses induced by EMF *in vitro*. A very large number of cellular components, cellular processes, and cellular systems can conceivably be affected by EMF. However, because evidence from theoretical and experimental studies suggested that EMF are unlikely to induce DNA damage directly, most studies have been conducted to examine its effect on the cellular membrane, general and specific gene expression, and signal transduction pathways.

Increases in ornithine decarboxylase activity in cultured mammalian cells, involved in the induction of tumur cell proliferation after exposure to EMF, were reported by Byus et al.[21] and Mullins et al.[22]. Uckun et al.[16] described the stimulation of protein tyrosine kinases, which are important in the initiation of second messenger cascades regulating cell proliferation, and pulsed EMF (PEMF) is described as a modulator in critical events in receptor-mediated signal transduction[14,15].

Experimental investigations have addressed the interaction between EMF and calcium fluxes because calcium is a principal regulator of several cellular processes. Modulations in intracellular $Ca^{2+}$ concentrations during exposure to EMF were reported by several investigators[23,24,25,26,27,28]. The authors concluded that modulation of intracellular $Ca^{2+}$ concentrations is possible only in prestimulated immune responding cells[13,29].

Also, alterations in DNA and RNA synthesis rates (for review [30]) were found to be produced by EMF[10,11,12,25]. However, Saffer and Thurston[31] found no alterations on myc-expression in HL-60 cells after short-term exposure to EMF.

Miyakoshi et al.[32] have shown that after exposure to very high-density magnetic fields (MFs) (400 mT), mutations in the hyopoxanthine-guanine phosphoribosyl transferase (HPRT) gene were induced in a human osteosarcoma cell line. A later study of these authors showed that the threshold for mutation induction has to be greater than 5 mT or 115 mA/m2 in Chinese hamster ovary K1 (CHO) cells[33]. Furthermore, an increase of X-ray–induced mutation rate was reported after exposure to X-irradiation (3

Gy) followed by a long-term exposure to 5 mT MF. Additionally, a dose-related increase in mutation frequency induced by MF (60 Hz, 0.47–0.7 mT) was reported after pre-exposure to γ-irradiation (2 Gy) by Walleczek et al.[34].

It has been suggested that cells respond to EMF via changes in transcription and translation of heat-shock proteins (hsp70 mRNA) or of heat-shock transcription factor (hsf1), as a reaction to an unspecific environmental stress[12,35,36,37]. However, it has been published by Parker and Winters[38] that exposure to rotating 60-Hz fields (0.1 mT for 24, 48, or 72 h) did not induce hsp70 mRNA synthesis in cultured mouse and human cells.

There is evidence that cell shape is a major determinant of differentiation[39] and is a marker of differentiation in different cell types. Alterations in cell morphology, size, and orientation can reflect cellular responses to EMF. In a series of investigations of Blackman's group, the effect of EMF on neurite outgrowth in a well-developed model of neuronal differentiation was studied[40,41,42,43]. PC-12 cells were primed for growth by plating them on collagen-coated dishes. Cells were exposed to 0–50 µT MFs and a neurite outgrowth suppression of about 20% was observed with a maximum effect after exposure to 10 µT MF.

Lacy-Hulbert et al.[44] observed no changes on gene expression after quantitative northern blot analysis of myc and beta-actin mRNAs from HL-60 cells exposed to 60-Hz MFs of 0.57, 5.7, or 57 µT for 20 min. However, it was shown by Santoro et al.[45] that after long-term exposure to EMF (2 mT for 72 h), components of the cytoskeleton were reorganized and membrane fluidity was modified in human Raji cells. In our study, no influence of EMF on the cytoskeleton and on motor proteins, both microtubule- and actin filament–based, was detected in murine macrophages under a short-term exposure regime of 1 mT for 4 h (Fig. 1), but also after a long-term exposure for 72 h[46].

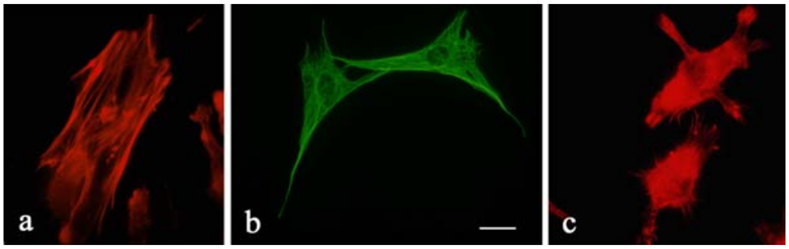

**FIGURE 1**. Immunofluorescence micrographs of cytoskeleton elements in mouse bone marrow–derived macrophages. (a) Rhodamine-phalloidin labeling of hepatitis virus–induced stress filaments, (b) microtubule labeling with monoclonal antibody, and (c) rhodamine-phalloidin labeling of actin filaments after EMF exposure to 1 mT for 72 h. No changes in the arrangement of cytoskeleton elements could be detected. Bar = 10 µm.

Intercellular communication has been postulated as a necessary condition for cells to progress through normal differentiation. Gap-junctional communication is considered to play a key role in tissue homeostasis and its disturbance has been associated with various health problems. Ubeda et al.[47] investigated changes in gap-junctional competence in dye-coupling experiments and they described a blocked upregulation of gap-junctional coupling caused by exposure to the field (1.6 mT). However, Schimmelpfeng et al.[48] showed that a 50-Hz field could upregulate intercellular coupling in dye microinjection studies with NIH 3T3 cells.

Overall, studies performed so far have not identified the underlying mechanisms inducing cellular responses to EMF. On the other hand, the experiments have shown that moderately intense fields can interact with various biological systems, which depends on biologic and on physical conditions. Thus identification of mechanisms is important in resolving both scientific uncertainty and potential public health issues.

## CELL BIOLOGICAL MECHANISMS AND THEIR POTENTIAL INVOLVEMENT

### Genotoxicity and Cytotoxicity

It is generally accepted that 50/60-Hz EMFs do not transfer energy to cells in sufficient amounts to damage DNA directly leading to genotoxic effects. However, it is possible that certain cellular processes altered by exposure to EMF, such as free radicals, indirectly affect the structure of DNA causing strand breaks and other chromosomal aberrations, including sister chromatid exchange, micronucleus formation, or effects on DNA repair, or leading to cytotoxic effects inducing cell death.

### Micronucleus Induction

Genotoxic effects after EMF exposure, such as micronucleus formation *in vitro*[49,50,51], chromosomal aberration[52] or sister chromatid exchange[53,54], and chromatid breaks[55,56,57] have been reported by different investigators. Lai and Singh[58] reported dose-dependent DNA strand breaks after using microgel electrophoresis, which is a very sensitive assay for genotoxicity, and Ivancsits et al.[59] confirmed these data. However, a number of other studies reported that EMF does not induce chromosomal damage *in vitro*[60,61,62,63]. The discrepancy of the observed data could be due to differences in the cell types used, exposure duration (from hours to days), and exposure conditions (e.g., orientation of electric- and/or magnetic fields, etc.). The magnitude of the induced electric fields ($E_{max}$) and the induced current densities ($J_{max}$) can vary depending on the size of the cell culture dish and/or height of the filled in medium, as mentioned before.

It was shown that MFs oriented vertically with respect to the surface of the culture medium induce micronucleus (MN) formation in a human squamous cancer cell line (SCL II) after long-term (24–72 h) exposure to 50-Hz 1-mT MF[49]. In a comparative study, MN induction was investigated in human amniotic fluid cells and a significant increase in MN induction occurred after horizontally applied MFs, whereas vertically generated MFs induced no genotoxic effects[50].

The obtained results show that the orientation of MFs and the physical condition of the exposure system are of major importance for the induction of genotoxicity in certain cell types. Therefore, the reason for inconsistent results published in the literature may well be caused by the variability of exposure systems, the exposure conditions, and the cell types used.

There are several possibilities to explain the findings that exposure to EMF induces genotoxic effects under certain exposure conditions. Tofani et al.[64] described MN induction in human peripheral lymphocytes after EMF exposure to 32 Hz only in the presence of the geomagnetic field, whereas no effect could be observed when the static geomagnetic field was nulled. They concluded that the conditions used elicited the modulation of intracellular $[Ca^{2+}]i$ influx, which has the potential to influence several cell functions, such as RNA, DNA, and protein synthesis and is involved in signal transduction pathways as well. Special physical conditions of electric and magnetic fields seem to be important to induce genotoxic effects by indirect mechanisms. The so-called "window effect" (for review [65]) was discussed in several studies[52,66,67] with evidence for intensity- and frequency-dependent cellular responses. Diener and Eberle[68] reported an increased MN induction after exposure to combined specific amplitude of $B_{AC}$ and $B_{DC}$, but not after $B_{DC}$ (1 µT/50 Hz) alone.

There is evidence that genotoxic effects after EMF exposure, as measured in the MN assay, must be induced by an indirect mechanism, e.g., magnetic fields-stabilized free radicals[69].

### Apoptosis/Cell Survival

Programmed cell death, also called apoptosis, is a physiological mode of cell death occurring in development and cell differentiation, in tumor cell depletion, and in response to mild damaging stimuli[70]. Apoptotic cells have well-defined morphologic characteristics, such as formation of cytoplasmic blebs (actin-dependent protrusions), “apoptotic bodies” in the nucleus (chromatin condensation at the nuclear periphery), and the nuclear fragmentation in membrane-bound vesicles (including digested DNA into 180- to 200-kbp fragments). Since the apoptotic process is very rapid and connected with a loss of cell-cell contact, the detection is limited microscopically. In order to detect the typical DNA-ladder formation electrophoretically after DNA-fragmentation, at least 30% of apoptotic cells is needed.

In a study, the induction of MN induction and apoptosis in SCL-II cells exposed to 0.1–1.0 mT 50-Hz magnetic fields was investigated using the criteria of morphological changes to detect apoptotic cells microscopically, since no strong induction of apoptotic cells was expected[49]. The cells showed a dose-dependent increase in MN frequency together with the increase in apoptotic cells after exposure to 50-Hz vertically applied MF (0.8 and 1.0 mT). In contrast, in nontransformed amniotic fluid cells, no significant changes were noted, suggesting that different cell lines react differently. On the other hand, it was observed that cells containing MN undergo cell death mainly by apoptosis. However, this phenomenon is not directly due to EMF exposure; it seems to be a general principle for cell death by cells containing MN. Ismael et al.[71] observed an increase in apoptosis of mouse thymocytes treated with dexamethasone using the specific assay system for apoptotic cells (TUNEL assay). The increase was observed only in thymocytes and not in splenic T-cells from animals exposed additionally to a 0.4–1.0 µT 60-Hz magnetic field.

### Cell Proliferation and Cell Cycle Control

#### *Cell Proliferation*

Cell proliferation is a very complex process that is under the control of multiple cell signal transduction pathways. Maintaining the integrity of genetic information during cell proliferation is fundamental for living systems. It is therefore vital for cells that DNA damage induced by spontaneous hydrolytic events or by radiation or chemical mutagens is effectively recognized and repaired efficiently. Unrepaired or inaccurately repaired DNA can lead to cell death (necrosis or apoptosis) as well as to genomic instability, mutations, and ultimately to cancer.

Altered proliferation of cells after EMF exposure has been observed in a number of studies *in vitro*. An increase in cell cycle progression of human lymphocytes exposed to 5 mT (50 Hz) were shown[54,72,73] and West et al.[73] demonstrated increased colony growth in anchorage-independent JB6 cells after 10–14 days exposure to 1.1 mT (60 Hz). In a study by Katsir et al.[74], an increase in cell proliferation with exposure over the frequency range of 50–100 Hz and intensity range of 0.1–0.7 mT was demonstrated. Both frequency- and intensity-dependent responses were observed with a maximum enhancement of proliferation of 70% after exposure to 100 Hz at 0.7 mT. A contradictory result was reported by Schimmelpfeng and Dertinger[75] who found a reduction in cell number after exposure to a 2-mT, 50-Hz field of SV40-3T3 cells.

#### *Cell Cycle Control*

DNA damage or incomplete replication of DNA results in inhibition of cell cycle progression at the G1/S or G2/M checkpoints by highly conserved regulatory mechanisms. The proliferation of eukaryotic cells is controlled at specific stages of the cell cycle by various cyclin/Cdk complexes, which can be induced in response to mitogenic signals. Their levels are determined by transcription as well as by precise proteolytic degradation with the fundamental role to integrate extracellular signals within the cell cycle.

Numerous cyclins have been identified, including cyclin A and A1; cyclin B and B2; cyclin C; cyclin D1, 2, and 3; cyclin E and E2; cyclin F; cyclin G1 and G2; cyclin-H; cyclin I; and cyclin T1 and 2; which are involved in cell cycle regulation (for review see [76]). Cyclin B/Cdc2 plays a critical role in the G2 to M phase transition, whereas cyclin A/Cdk2 dominates in S phase. Cyclin A1 associates with Cdc2 and to a lesser extent with Cdk2. The cyclin D family members regulate Cdk4/6-mediated phosphorylation of the retinoblastoma protein (Rb) in middle to late G1 phase which leads to release of active transcription factors required for entry into S phase[77]. Cyclin E is involved in the regulation of Cdks at the beginning of S the phase. Cyclin F expression peaks in the G2 phase, whereas cyclin G2 is highly expressed in the late S phase, and cyclin G1 expression is constitutive.

Cdks are serine/threonine kinases and critical regulators of cell cycle progression, and are constitutively expressed throughout the cell cycle by selective phosphorylation and dephosphorylation at specific amino acid residues[78,79]. Cdk activity is tightly controlled by subcellular localization[80] and the association of Cdk with a particular subset of cyclin and Cdk inhibitors (CKIs)[81,82].

The activities of Cdks are controlled by, both positive regulator cyclin and negative regulators, CKIs to induce cell cycle arrest in response to different signals as well as through phosphorylation of the Cdks themselves[83]. Two families of CKIs have been shown to play a major role in regulating orderly progression through the cell cycle. Members of the INK4-family (INK4 = INhibitors of cdK4) include $p16^{ink4a}$ and $p15^{ink4b}$, $p18^{ink4c}$ and $p19^{ink4d}$, which specifically inhibit Cdk4/6 and prevent formation of active cyclin D/Cdk complexes[84,85]. The second family includes the CIP/KIP-family, $p21^{Cip1}$, $p27^{Kip1}$, $p57^{Kip2}$ and they inhibit a range of Cdk activities[86]. These proteins bind to Cdks and inhibit the catalytic activity of the Cdk-cyclin complexes. In particular two of these CKIs, $p16^{INK4a}$ and $p21^{CIP1}$, have been implicated as potential tumor-suppressor genes in cancer[87,88].

In a study of Eremenko et al.[89] and in other studies of Lange et al.[90,91] alterations in the G1 phase was reported due to exposure to 50-Hz EMF, which were focused on cell cycle regulating proteins on cyclin D1, Cdk4, $p16^{INK4a}$, and $p21^{CIP1}$. Each of these proteins is important for the progression through the G1 phase and the G1/S transition and seems to play a critical role in cancer development.

### The G1 Phase

During the G1 phase, cells require extracellular signals up to the restriction point (R). R is close to the end of the G1 phase, prior the entry into S phase. Signals can be growth factors, polypeptides secreted by cells that are recognized by other cells. EMFs may possibly act as such a signal. Growth factor receptors are single transmembrane proteins, receptor tyrosine kinases (RTKs), containing tyrosine kinase domains in the cytoplasmic portion of the receptor. After binding growth factors, the RTKs dimerize and transphosphorylate critical tyrosine residues in the cytoplasmic portion of the receptor. These phosphorylated tyrosines act as docking sites for proteins and induce a cascade of signal transduction events to activate various transcription factors followed by activating transcription of responsive genes such as the cyclin D gene.

Cyclin D binds to Cdk4/6 and phosphorylates Rb, which is a classic tumor-suppressor protein. Rb binds to a family of transcription factors (E2F) and represses transcription of E2F-responsive genes during G1 phase (Fig. 2).

The cyclin E/Cdk2 complex binds to the C-terminus of Rb and phosphorylates the middle part of the protein. E2F is then released, which initiates the activation of the transcription of E2F-dependent genes to start G1/S transition. Cyclin E levels are regulated by Rb-E2F; when cyclin D/Cdk4/6 phosphorylates Rb, this leads to a partial inactivation, followed by cyclin E transcription. Cyclin E complexes with Cdk2 resulting in the additional phosphorylation of Rb and subsequently to the complete inactivation by releasing E2F[76]. Following stress conditions, the INK4 CKIs are expressed and prevent Rb phosphorylation. The Cip/Kip family of CKIs bind and inhibit cyclin E/Cdk2 by inhibition of Cdk activity, which prevents its ability to stimulate cell cycle progression. While p21 levels are induced during periods of stress, p27 is constitutively expressed when the cell is quiescent (G0 and G1 phases) and is significantly reduced at R and remains low until the end of the M phase.

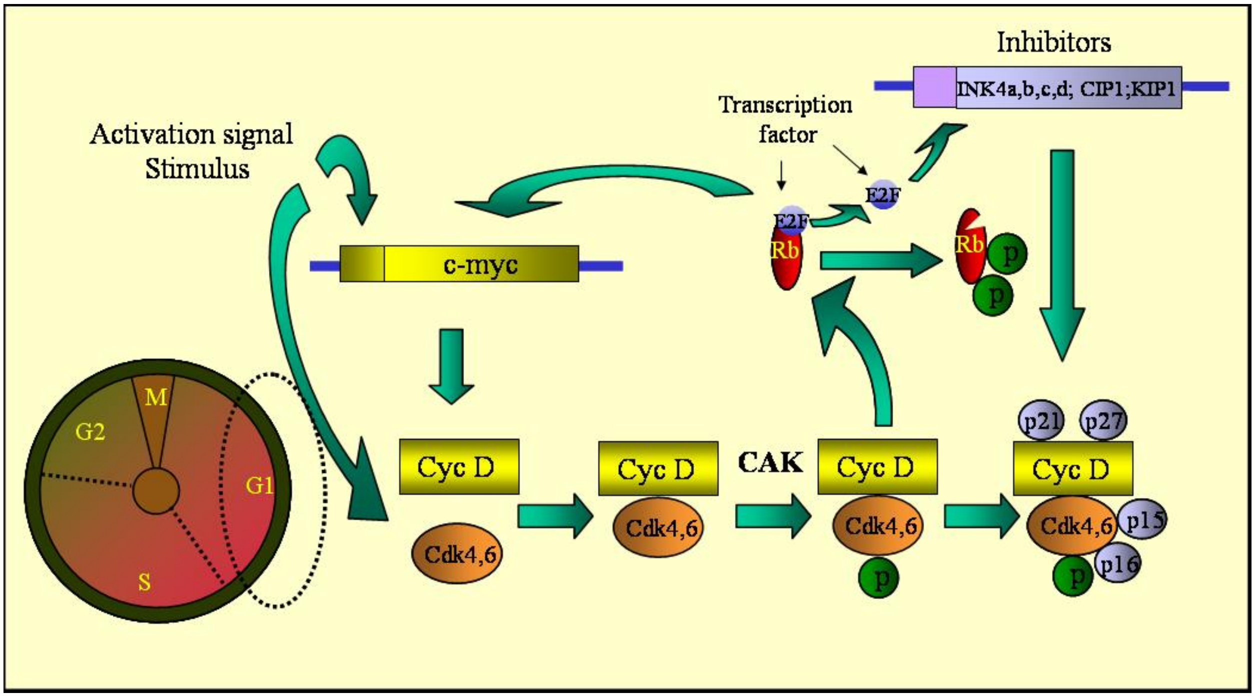

**FIGURE 2.** The G1 phase: cyclin D/Cdk4 or Cdk6 phosphorylate and inactivate Rb releasing E2F (which stimulates the transcription of genes necessary for cell cycle progression). INK4 CKIs bind only to Cdk4/6, inhibiting their activation by cyclin D, while Cip/Kip CKIs are acting as assembly factors for cyclin D and Cdk4/6. These CKIs inhibit the phosphorylation of Rb and thus leads to cell cycle arrest in G1.

In order to elucidate the molecular principles of the lengthening of G1 phase, the expression patterns of several G1 regulatory proteins were examined by Lange et al.[90,91]. Cyclin D1 and its catalytic partner, Cdk4, were investigated because there is evidence to suggest that variations in the expression of both result in modifications of the G1 phase. The results support the suggestion that exposure to EMF causes no changes in the protein levels of Cdk4 and in addition no alterations in the expression pattern of cyclin D1 were detected. These data are in accordance with results reported by Dees et al.[92], where no evidence was found to show that cyclin D1 synthesis was altered in MCF-7 cells exposed to 60-Hz EMF at 9 G for 6, 9, and 12 h.

The analysis of cell cycle inhibitory proteins showed that EMF led to a weak increase in the level of $p16^{INK4a}$ during exposure to EMF[90,91]. It seems likely that the EMF-induced alterations in the level of $p16^{INK4a}$ mainly influence the regulation of Cdk4 in amniotic fluid cells. Numerous studies showed that increasing amounts of $p16^{INK4a}$ can effectively block the cyclin D1/Cdk4 interaction[86,93] which in turn leads to an inhibition of Cdk4 kinase activity. This generally causes cells to accumulate in G1 and prevents them from entering S phase[94]. The enhanced expression of $p16^{INK4a}$ after EMF exposure is in accordance with the observed retardation of DNA synthesis[90]. In the same study, an increased level of $p21^{CIP1}$ was detected after EMF exposure, which is in agreement with these findings since $p21^{CIP1}$ is able to inhibit the Cdk4 kinase activity and hence influence the G1/S transition as well. However, $p21^{CIP1}$ is a general inhibitor of several cyclins and Cdks[95]. Therefore, the observed delay in the G1 phase may also be a consequence of the interaction of $p21^{CIP1}$ with cyclin E/Cdk2 complexes, which play an important role in regulating the transition from late G1 to S phase[91].

### The S Phase

During the synthesis phase, cells replicate their DNA to allow the division of cells into two daughter cells, each with a complete copy of the genome.

Since exposure to low-frequency EMF might cause changes in the DNA synthesis, BrdU incorporation during DNA synthesis can be used to detect perturbations in cell proliferation. A number of studies have shown the proliferative effect of EMF with regard to specific cell cycle parameters. In general, these data are consistent with a small decrease in the rate of progression through the early part of the cell cycle. Cridland et al.[96] revealed that exposure to weak MF (50 Hz, 20 µT and 200 µT) led to a delay in the onset of DNA synthesis in synchronized normal human fibroblasts, which indicates a lengthening of the G1 phase. Schimmelpfeng et al.[75] reported that exposure to 50 Hz, 2 mT caused alterations of several indicators of cell proliferation, including an accumulation of G1 phase cells. However, in other studies, no or opposite effects were found. Zhao et al.[97] failed to demonstrate any significant differences in the rate of DNA synthesis after EMF exposure (60 Hz, 0.1–0.8 mT) in INIT/10T1/2 cells. Results of Wei et al.[98] suggest that 60-Hz magnetic fields at intensities of 0.9 and 1.2 G, respectively, can increase [3H] thymidine incorporation into DNA of astrocytoma cells in a time-dependent manner, while 0.6 G had no effect on the DNA synthesis. Lange et al.[90] showed that exposure to MFs (1 mT) modifies the passage of amniotic fluid cells through the cell cycle, leading to a retardation of DNA synthesis.

Taken together, these results show the wide variety of effects of EMF exposure regarding cell proliferation; reaction of different cell types are dependent on cell age, differentiation stage, activation, and/or metabolic state, which have to be taken into consideration[67,89,96,99,100,101]. Moreover, the fact that most of the observed effects are of a very small magnitude (often in the order of only 10–20%) additionally complicates the evaluation of the controversial results and the verification of published studies.

### The G2/M Phase

Disturbances in the G2/M-phase of the cell cycle are expected when DNA strand breaks and/or chromosome breaks are involved in genotoxic effects, and such damage would result in an altered growth curve as well as in an altered mitotic index. In studies of the influence of EMF on events characterizing, in particular, the G2 and M phase of the cell cycle, neither a significant change in the rate of DNA repair (which is a hallmark of the G2 phase of the cell cycle), nor an effect on the mitotic activity (reflecting the M phase) could be observed[50,102,103]. Accordingly, BrdU incorporation involved in DNA repair during the G2 phase can be ruled out. Similar outcomes were described in a study by Nindl et al.[100]. They reported that treatment with different kinds of EMF (1.8 mT; 60-Hz field, 0.1 and 0.4 mT) diminished the extent of [3H] thymidine incorporation into DNA of Jurkat cells and that this effect occurs only after the completion of several cell cycles. The authors concluded that the EMF effect was not due to inhibition of DNA repair.

### Signal Transduction/PKC

Signal transduction occurs in cellular systems at the cell membrane and inside the cell, where signals from the environment and from other cells are received and processed. These signals regulate intracellular processes, such as metabolic activity, gene expression, differentiation, and cell proliferation. Signal transduction processes are important pathways by which EMF may affect cell function. Membrane-mediated signal transduction by hormones and other signaling agents involves transmission across the plasma membrane that does not require penetration of the membrane. Signals associated with a conformational change of a membrane protein are propagated across the cell membrane by three well-understood mechanisms: opening and closing of ion channels and resultant current flow; changes in an intrinsic enzymatic activity of the receptor; and changes in the affinities of the receptor for intracellular proteins, which might have enzymatic activity.

In almost all cases, the mechanism of signal transduction distal to the receptor involve intracellular pathways due to changes of the ionic composition of the cytosol (e.g., changes in intracellular calcium) or changes in phosphorylation of intracellular proteins (e.g., enzyme activity, enzyme regulators, and transcriptional regulatory factors). Cellular responses to signals are either short term, with little or no persistence after removal of the signal, or long term, involving persistent changes in the function of cells, such as increased or decreased proliferation, changes in gene expression or differentiation, and, in some cases, apoptosis. The short-term changes are generally mediated by modification of enzyme activity in the cytosol or of the membrane of the cell. The long-term changes invariably involve alteration of nuclear function, such as transcription, cell-cycle regulation, and cell division.

Members of the PKC family play a key regulatory role in a variety of cellular functions including cell growth and differentiation, gene expression, and hormone action. PKCs were originally identified as protein serine/threonine kinases whose activities are dependent on calcium and phospholipids.

It was reported that phorbol esters exert their tumor-promoting effects by activation of PKC[104]. When PKC is activated either naturally or synthetically, the cytosolic enzyme will associate with the cell membrane. In a series of experiments, no translocation of PKC could be observed by Western blot analysis after exposure to EMF in amniotic fluid cells, but EMF in combination with the tumor-promoting phorbol ester TPA markedly alters the subcellular localization of PKC[18]. Tao and Henderson[105] described an additive effect of both 60-Hz EMF at 1 G and TPA treatment on differentiation of HL-60 cells at low TPA concentrations, whereas higher concentrations eliminate this effect because of TPA "saturation". Similar data published by Tuinstra et al.[106] indicate that EMFs may be acting in a synergistic manner with low levels of TPA on PKC activity. The authors observed an EMF effect only in cells that are moderately activated by 50-nM TPA, but not in cells that are unexposed or treated with higher concentration (2 µM) of TPA. It seems that cells exposed to suboptimal concentrations of TPA shift from a normal into an activated state without saturating the physiological capacity to respond to TPA, so allowing an additional effect caused by magnetic field exposure.

There are several reports about the influence of electric and magnetic fields on PKC activity or other kinases involved in signal transduction cascades in different cell systems. It has been shown that treatment of HL-60 cells with 60-Hz electric fields result in decreased cytosolic, but unchanged membrane-associated PKC, activities[17]. Phillips et al.[107] showed an increase in PKC activity on exposure to EMF. Uckun et al.[16] indicated that 60-Hz 1-G fields caused activation of tyrosine kinases and PKC in human pre-B-leukemia cell line. A study of Dibirdik et al.[108] demonstrated that exposure of DT40 lymphoma B cells to EMF results in a tyrosine kinase–dependent activation of phopholipase $C\gamma 2$ leading to increased inositol phospholipid turnover. One may speculate about the mechanism by which EMF affect enzyme activity[109], but it can be suggested that cells become more susceptible to effects of MF exposure because of their sensitivity to TPA.

### Immune Cell Activation

Cell regulatory processes, involving the induction of free radical production that are able to interact with DNA or other cellular components, can lead to a potentiation of free radical–dependent effects[110] and are, therefore, assumed to be a more likely target of EMF-induced cellular responses. Macrophages play an essential role in the body's defenses and immune system. Activated macrophages release free radicals as reactive oxygen species (ROS), reactive nitrogen species (RNS), and also cytokines and are associated with the onset of phagocytosis. ROS are unstable reactive molecules that are produced continuously in several cells. Free radicals including superoxide anion radicals, hydroxyl radicals, and hydrogen peroxides are formed as by-products in various metabolic processes. ROS are involved in intracellular signal transduction pathways and regulation of gene expression determining the anti-inflammatory response, cell growth, differentiation, proliferation, and stress response[111]. Superoxide radical anions are the precursors of additional ROS. The formation of superoxide radical anions is mediated by enzymes such as the NAD(P)H oxidase, xanthine oxidase, and the nonenzymatic redox reactive compounds of the mitochondrial respiratory chain constituting the primary defense mechanisms[112,113,114]. Activated phagocytic cells produce a large amount of ROS via the NADPH oxidase, whereas nonphagocytic cells produce it through the induction of NADH oxidase leading to a lower level of free radicals. The activation of NAD(P)H oxidase can also be stimulated by the receptor-independent acting agent TPA or by the receptor-dependent activator LPS. TPA activates the PKC, which phosphorylates $p47^{phox}$ and activates the small G protein Rac through the mitogen-activated protein kinase (MAPK) pathway[115]. High-level production of free radicals in the organism has shown an increased potential for cellular damage of substances such as DNA, proteins, and lipid-containing structures. In contrast to molecules such as cytokines (large molecules signaling by docking with specific receptors and change molecular surfaces on the target cells), molecules such as ROS could react with diverse cell compounds in a nonspecific mechanism. Therefore, free radicals play a decisive role in cytotoxicity and also as cellular messengers to control noncytotoxic physiological responses.

EMF has been shown to increase ROS levels in phorbol ester–activated neutrophils[116], but it was unclear whether in nonactivated macrophages short-term EMF stimulation would elevate ROS levels via stimulation of the physiological mechanisms.

It was shown that 1-mT MF is able to activate macrophages and to increase the internalization rate to about 135%[46]. In a previous study[117] using prestimulated cells, a decrease in phagocytic uptake of beads combined with increased intracellular $Ca^{2+}$-levels was reported. However, in this study, cells were exposed to static and much higher (25–106 mT) magnetic fields, as well as for prolonged periods of time (24 h).

It is not known why various cell types respond to EMF with different efficacy, but it seems to be clear that several cell types are responsive to EMF by different mechanisms. The responsiveness might well depend on the number and the distribution of cell-specific membrane receptors, which are variably expressed on different cell types. It is known that TPA enhances the stimulation of macrophages for phagocytosis via the phospholipid-dependent PKC pathway by imitating diacylglycerol[118]. It is noteworthy that the TPA effect on differentiation of HL-60 cells was reported to be mimicked by EMF[105], similar to the TPA effect on activation of macrophage phagocytosis[46].

Phagocytosis in activated macrophages is known to be associated with the production of superoxide radicals. A significantly increased superoxide anion radical production in the absence of phagocytic activity was detected after exposure to EMF in mouse macrophages[46,119] and also in human monocytes[120], which is a clear indication for cell activation. These investigations demonstrate a low, but significant, induction of free radical release indicating a specific activation of the NADH-oxidase after EMF exposure. On the other hand, the activation of NADPH-oxidase would result in a higher production of free radicals leading to an oxidative burst. Roy et al.[116] reported an increased oxidative burst after exposure to 0.1-mT MF in TPA-activated rat peritoneal neutrophils and the authors proposed an increased lifetime of intracellular free radicals due to the field exposure.

### Initiation or Promotion: Steps in Carcinogenesis

Animal studies have shown a possible copromoter capacity of EMF on tumor growth[121]. *In vitro* studies showed biological effects after exposure to EMF, such as increased colony growth in anchorage-independent JB6 cells[73]. Cain et al.[122] showed that exposure to EMF combined with treatment of a chemical tumor-promoter phorbol ester (TPA) enhances cell transformation *in vitro*. These data suggest that EMF and chemical tumor promoter are able to act as copromoters, which may lead to tumor enhancement in initiated cell population.

It has been reported that EMF exposure alone did not cause MN formation in syrian hamster embryo (SHE) cells, whereas after treatment with a tumor initiator benzo(a)pyrene (BP), the MN frequency was increased[103]. The tumor-promoter TPA or EMF alone caused neither increased MN formation nor changes of the mitotic activity in exposed SHE cells. Double treatment experiments with EMF and TPA or EMF and BP, as well as in reversed order, was used to test for the presence of initiating or cocarcinogenic (“promotion-like”) potency of EMFs, but no differences in MN formation were found. Interestingly, an increased MN rate was detected when cells were pre-exposed to EMF and then treated with BP under EMF exposure and exposed again to EMF. This finding suggests that EMFs can enhance the tumor-initiation process of BP. Similar “coinitiating” effects were reported by Bowden and Rosenthal[123], who showed that caffeine is able to enhance DNA initiation in mouse primary epidermal cells treated with BP. Kim et al.[124] found that BP-initiated MN formation is coupled with peroxidase-dependent “bioactivation”, resulting in DNA oxidation by the release of ROS. Therefore, an increased metabolic activation process caused by EMFs seems to be the reason for elevated initiation effect[96,100,103]. It was proposed that TPA might act indirectly by the release of free radicals[125,126]. Additional treatment with a “cellular activator” such as EMF may well be the reason for an increase in genotoxicity. Treatment of cells with a carcinogen, such as BP, TPA, and also EMFs, may cause an “activated state” of the cell and therefore enhance the rate of genotoxic events by the elevated release of ROS. Moreover, EMFs were reported to interact with some compounds of the signal transduction pathway[13,17]. This is in analogy to TPA, which interacts directly with PKC leading to an induction of the PKC-mediated pathway. It is also known that nonphysiological or unusual extended stimulation of signal transduction mechanisms may result in genetic instability[127].

Likewise, it is possible that magnetic fields are able to increase genotoxic effects via indirect mechanisms, such as the release of reactive intermediates and/or metabolic cell activation processes, leading to genetic instability and cell cycle disturbances.

McLean et al.[128] reported that SENCAR mice pretreated with the tumor initiator DMBA and exposed to 2-mT EMF did not show skin tumor development. This finding was confirmed by Rannug et al.[129,130]. A significant increase in tumor development could be observed in studies after additional application of TPA in these mice[131,132] or with modified time schedules of chemical application to rats[133,134,135]. As Juutilainen et al.[136] stated in a review, current *in vivo* experiments on the possible carcinogenecity of EMF are inadequate for environmental exposure of carcinogens to humans when common experimental initiator/promoter design was used. Since environmental exposure of the general public may well comprise several types of carcinogens, including continuous exposure to a wide spectrum of EMFs, it seems important to note that the exact time point of initiation during continuing EMF exposure may be of crucial importance.

### CONCLUSION

There are many reports published to elucidate possible correlation between EMF and cancer or other diseases, but no causal relationship is supported by the discussed mechanisms. Several physical possibilities of interaction are discussed[137], not included in this review, but it seems that the interaction between EMF and cellular systems is not based only on one of the described mechanism. Moreover, a multiplicity of triggering of transitions seems to amplify cellular reactions, causing different effects, which can depend also on the affected cell types. Externally applied fields are able to shift the equilibrium distributions of ions slightly, as described by Blank[138] in his surface compartment model. Small deviation of the local concentrations near membrane-associated enzymes and proteins may trigger their function and induce connected cascades.

The combination of different biological conditions and physical parameters may result in “multiple windows” that interact by secondary mechanisms causing cellular responses that lead to genotoxicity or other cellular changes. The cell shape seems to be also of importance because the induced flux density can be focused in different regions on the cell surface or within the cell causing “hot spots” with very high electric field intensities. It is known that local deposition of energy within cells induce genotoxic and cytotoxic effects depending on the localization to the target molecules, shown in studies using Auger electron emitter. Auger electrons are characterized by a very small energy leading to a very high energy density in a rather small volume and act with various effectiveness in different cell types[139,140].

The induced field densities or magnetic fields can influence enzymatic activities by mimicking receptor binding, which affects proteins and other biomolecules to activate biochemical cascades and "out of schedule" cellular reactions. These induced physiological reactions cause cellular effects through secondary or indirect mechanisms such as cell activation processes, probably through the release of free radicals and/or the out of schedule switching-on of transduction pathways.

It has been hypothesized by Stevens[8] that exposure to 50/60-Hz EMF may suppress the nocturnal production of the pineal hormone melatonin. A later report suggests that reduced levels of melatonin could lead to an increased risk of breast cancer[141]. A number of studies have been done to elucidate the role of melatonin and the mechanism of its physiological activity. Melatonin acts as a receptor-independent free radical scavenger and antioxidant, and is able to reduce the incidence of mutations and likelihood of developing cancer. Moreover, melatonin inhibits the growth of different types of established tumors[142,143]. It seems that melatonin inhibits mammary tumor genesis in animals and *in vitro*[144,145] and acts in an oncostatic manner in different cancer cells[143]. Several studies described reduced levels of melatonin in EMF-exposed animals[146,147] as well as *in vitro*[148,149,150], but short-term exposures to human volunteers failed to provided evidence for EMF effects on melatonin[151,152]. However, epidemiological studies reported suppressed levels of melatonin in occupational or residential EMF-exposed humans[153,154]. It remains to be determined whether EMF exposure is able to reduce the melatonin synthesis or its physiological effects to levels where an increased cancer incidence would be a consequence.

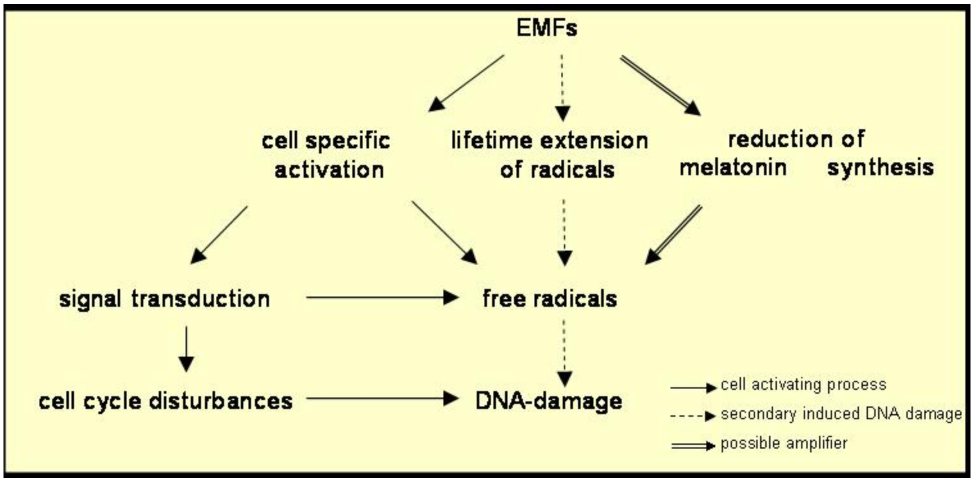

**FIGURE 3.** Different cellular pathways concerning free radical production after EMF exposure can lead to DNA damage. EMF exposure induces cell-specific activation processes, the induction of signal transduction pathways, and cell cycle alterations, which themselves induce free radical production. On the other hand, DNA damage can be induced due to secondary pathway(s), namely through the extension of free radical lifetime. Increase of free radical release can be also reached through the suggested suppressive capacity of EMF on melatonin synthesis. The reduced level of melatonin could function as a possible amplifier leading to an increased amount of free radicals. Altogether, EMF-induced reactions or cascades could lead to a higher incidence of DNA damage and, therefore, to an increased risk of tumor development.

Taken together, the question of a possible correlation between cancer and EMF has to be elucidated. Many effects are described, however, currently no convincing mechanism exists to provide a clue for estimating EMF-induced health risks. The different pathways of ROS involvement in physiological and pathological reactions after EMF exposure are summarized in Fig. 3. This assumption how cancer can be induced or promoted is an example for cell activation. It is obvious that EMFs are not able to induce genotoxic effects by directly, but due to indirectly mediated pathway(s). Cell shape and the origin are important and cell-specific membrane receptors seem to play a key role in the effectiveness to EMF leading to the activation of cell-specific actions. Cells respond specifically in dependence of their physiologic function and not in a general way, leading to the induction of cell type–specific signal transduction pathways and possibly cell cycle alterations (Fig. 3). Free radicals are important intermediates in physiological processes and are released during cell metabolism. It has been proposed that MF is able to stabilize free radicals in such a way as to increase their lifetime. If so, a prolonged lifetime of free radicals can increase the probability of radical-mediated DNA damage due a secondary induced mechanism. The so-called melatonin hypothesis describe that EMF suppress the level of melatonin synthesis; this reduced level of melatonin could function as a possible amplifier leading to an increased cancer risk.

Based on several lines of investigations, it is suggested that EMF exposure is able to perform cell type–dependent activation by means of increasing levels of free radicals. Those EMF-induced reactions could lead to a higher incidence of DNA damage and, therefore, to an increased risk of tumor development. On the other hand, these findings have also potential implications of short-term EMF exposure for therapeutic applications that, however, should be further explored.
